# Pressureless Mechanical Induction of Stem Cell Differentiation Is Dose and Frequency Dependent 

**DOI:** 10.1371/journal.pone.0081362

**Published:** 2013-11-21

**Authors:** Roland Fuhrer, Sandra Hofmann, Nora Hild, Jolanda R. Vetsch, Inge K. Herrmann, Robert N. Grass, Wendelin J. Stark

**Affiliations:** 1 Institute for Chemical and Bioengineering, ETH Zurich, Zurich, Switzerland; 2 Institute for Biomechanics, ETH Zurich, Zurich, Switzerland; 3 Institute of Anaesthesiology, University Hospital Zurich, Zurich, Switzerland; Northwestern University, United States of America

## Abstract

Movement is a key characteristic of higher organisms. During mammalian embryogenesis fetal movements have been found critical to normal tissue development. On the single cell level, however, our current understanding of stem cell differentiation concentrates on inducing factors through cytokine mediated biochemical signaling. In this study, human mesenchymal stem cells and chondrogenesis were investigated as representative examples. We show that pressureless, soft mechanical stimulation precipitated by the cyclic deformation of soft, magnetic hydrogel scaffolds with an external magnetic field, can induce chondrogenesis in mesenchymal stem cells without any additional chondrogenesis transcription factors (TGF-β1 and dexamethasone). A systematic study on the role of movement frequency revealed a classical dose-response relationship for human mesenchymal stem cells differentiation towards cartilage using mere mechanical stimulation. This effect could even be synergistically amplified when exogenous chondrogenic factors and movement were combined.

## Introduction

**Figure 1:**
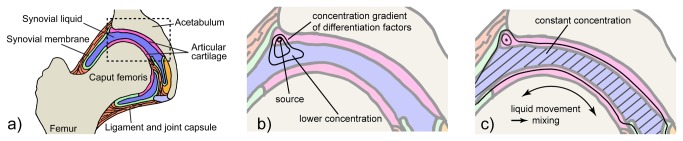
Concentration gradient distortion schematically shown in a joint Hip joint (anatomy, a) with a local concentration profile of a differentiation factor secreted at the interface of the cartilage if the joint is not moved (hypothetical, b) and under physiologic movement (c). Diffusion alone usually results in rather steep concentration gradients (static situation). Movement induces liquid mixing through convection (synovial fluid) flattening concentration profiles.

Chemical signaling organizes the structure of biological tissues[1] and stem cell differentiation during growth or repair. Diffusion, local release of growth factors and concentration gradients shape an organism’s 3D structure. Unfortunately, liquid filled pockets and particularly movement destroy such local information pattern (e.g. in a joint, see Figure 1) and challenge our present understanding of static cell-cell assemblies’ signaling[2,3]. 

We therefore hypothesize that soft movement is a key local tissue structuring factor. The role of movement has probably been best studied in tissue engineering for the growth of articular cartilage in vitro[4-8] using mechanical top load (i.e. pressure)[9-11]. Chondrocyte growth has been associated with demanding physical input such as cell deformation[12], hydrostatic pressure gradients[13], fluid flow[14], streaming currents[15] and physicochemical changes[16,17]. Similarly, substrate strain has also been used to improve hMSC differentiation into vascular smooth muscle cells for vascular tissue repair[18]. Clinical evidence provides a clear argument for the necessity of movement in function-guided, local cell differentiation[19,20]: Mammalian embryogenesis is characterized by an early start of intense movement (for humans, typically in the first third of gestation), a prerequisite to normal organ development[21] and takes place in a pressure-free environment. A second piece of evidence comes from the required absence of movement for healing bone fractures. Without adequate traumatic surgery or fixation, pseudoarthrosis may occur at non-fixed bone fracture sites[22]. Continued movement of such non-fixed primary callus then promotes local stem cell differentiation into cartilage and ligament tissue instead of bone tissue. A third argument arises when normal movement is impeded. If a joint is not moved anymore (e.g. due to arthritis), even an otherwise fully functional joint stiffens (ankylosis) and the tissue locally transforms into a bone-type material[23]. A fourth argument is counter-intuitive: Mechanical stress on ligaments regeneration has recently been shown by Altmann et al [24] to improve the healing process.

These clinical facts underline the importance of movement (Figure 1) and suggest that movement may follow a dose/response type relationship, similar to classical chemicals. Static mechanical properties (porosity, matrix elasticity/stiffness) have recently emerged as key factors in cell lineage specification[25,26]. In order to test our hypothesis, we took normal embryonic movement frequency as a starting dose[21] to run an in vitro study on the role of movement as a differentiation factor. We used pressure-free, soft and smooth deformations of a magnetic hydrogel scaffolds by time-programmed application of external magnetic fields. Human mesenchymal stem cells (hMSC) and chondrogenic differentiation were chosen as representative examples[27-29]. 

We demonstrate that purely mechanical stimulation provokes correct differentiation even in vitro and in the complete absence of any exogenous biochemical differentiation factors. This shows that mechanical stimulation is a key local organization factor at the cellular level. In a second series of experiments, we demonstrated that the frequency of stimulation and overall number of applied stimulations follow a dose-response relationship for these stem cells. This is similar to the present (biochemical) understanding where specific concentrations of the differentiation factor TGF-β indeed induce chondrogenesis[30] to a specific level of differentiation[31].

## Materials and Methods

Detailed methods on nanomagnet functionalization, characterization of functionalized nanomagnets and magnetic hydrogel, cell isolation, expansion and characterization, biochemical analysis and immunohistochemistry are provided in the Information in File S1.

### Magnetic hydrogel synthesis

**Figure 2:**
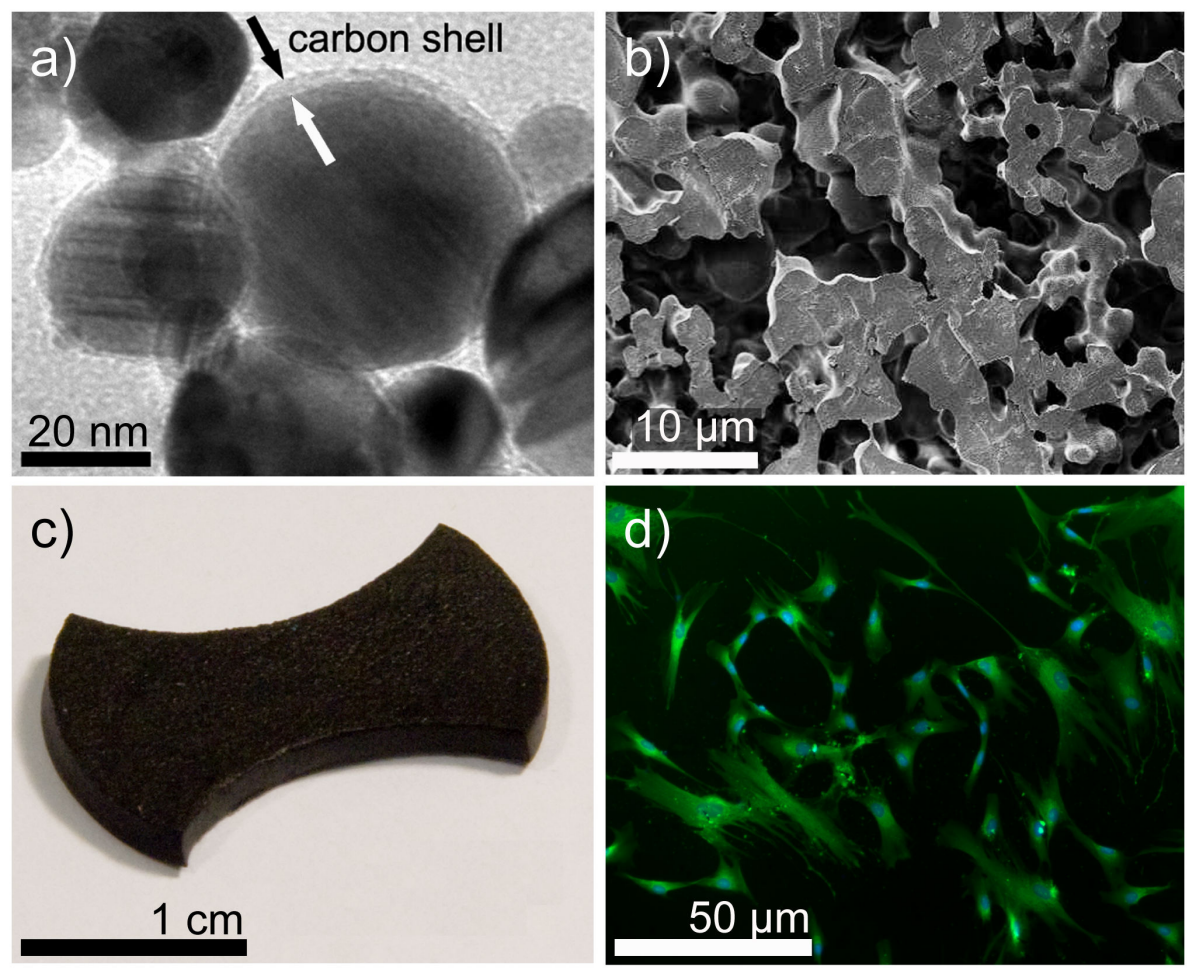
Magnetic and soft scaffold preparation and human mesenchymal stem cells. a) Carbon protected metal nanomagnets (transmission electron microscopy image) were covalently linked into a hydrogel (b, cryo-section) with high porosity (pore size ~ 10 µm) to facilitate cell attachment. c) Magnetic, soft, cell culture scaffold. The dog-bone shape minimizes the required magnetic force for soft deformation. d) Good adherence of human mesenchymal stem cells seeded on hydrogel surfaces (Calcein-AM staining).

Appropriate amounts of 2-hydroxy-ethyl-methacrylate (HEMA, 5.1 mL, puriss. ≥99%, Fluka), ethylene glycol dimethacrylate (EGDMA, 4.9 µL, purum ≥97%, Fluka) and styrene maleic anhydride copolymer (SMA® 1000H, 0.75 g, 36%, Sartomer) solution were mixed in water (25 mL, Millipore). Subsequently, 4-vinylbiphenyl functionalized nanomagnets (5 g) were suspended in the mixture using an ultrasonic probe (UP400S, 24 kHz, Hielscher GmbH) during 5 min. After addition of a rheology additive (BYK® 420, 0.782 g, Brenntag Schweizerhall AG), tetramethylethylenediamine (TMEDA, 31.9 µL, 99%, Sigma Aldrich) and ammonium persulfate (APS, 205 mg, 99+%, DNAse, RNAse and protease free, for molecular biology, Acros Organics) the mixture was processed again with the ultrasonic probe for 5 min. The reaction mixture was then poured into an electrophoresis casting mold (gel casting mold, kuroGEL Midi 13) and reacted for 1 h at ambient temperature. The obtained gel was treated with deionized water for 24 h while the water was changed 3 times. The procedure was repeated with phosphate-buffered saline (PBS, pH 7.4, GIBCO) to remove unreacted monomer and nanomagnets and to obtain a stable swelling behavior of the hydrogel. The obtained deep-black magnetic hydrogel (2 mm thickness) was punched out to the desired shape and sterilized in 70% ethanol. Sterile scaffolds were rinsed 3 times with fresh PBS to remove any residual ethanol. A dog-bone like shape of the scaffold (Figure 2c) was chosen to increase the flexibility and enable a hammock like deformation. This structure allowed for minimization of the required magnetic force.

### Scaffold seeding

A homogeneous distribution of seeded cells onto the hydrogel surface was obtained by using small volumes of relatively high concentrated cell suspensions (2.4•10^6^ P3 hMSC mL^-1^) either in control (DMEM, 10% FBS, 1% antibiotic/antimycotic) or chondrogenic medium (DMEM, 1% antibiotic/antimycotic, 100 nM dexamethasone (water-soluble, cell culture tested, Sigma-Aldrich), 6.25 µg mL^-1^ insulin (bovine pancreas, Sigma-Aldrich), 50 µg mL^-1^ ascorbic acid (≥95%, Sigma-Fine Chemicals), 40 µg mL^-1^ L-proline (≥98.5%, Sigma-Fine Chemicals), 6.25 µg mL^-1^ ITS (BD ITS+ Premix, BD Biosciences) and 10 ng mL^-1^ transforming growth factor beta 1 (rh TGF-β1, R&D Systems, [30]). Precut and sterilized hydrogel scaffolds were fixed with the aid of sterile stainless steel rings in wells of a 12 well plate (non-tissue-culture-treated, Falcon). The lower ring acted as a spacer with 2 mm thickness to allow hydrogel deformation. 250 µL of the respective cell suspension was seeded onto each scaffold and cells were left for 20 min at 5% CO_2_ and 37°C to allow adherence on the scaffold surface. An additional 1.75 mL of the respective medium was then gently added to each well. All cell culture experiments were performed in triplicate and the corresponding medium was replaced three times a week. 

### Cell attachment and viability

Successful cell seeding and adherence was verified by using 4 mM calcein acetoxymethyl ester (calcein AM, Invitrogen) in the corresponding medium for 20 min in a humidified incubator (37°C, 5% CO_2_). Fluorescence images from cells cultivated on scaffolds were immediately obtained using an inverted research microscope equipped with reflected fluorescence system (IX51, Olympus). Cell cytotoxicity was directly measured from the supernatant using a cytotoxicity detection kit measuring the lactate dehydrogenase (LDH) activity according to the manufacturer’s instructions (Roche Applied Science). 

### Cyclical magnetically assisted mechanical stimulation

The vertical motion of the magnetic, soft hydrogel scaffold was controlled by a magnetic field (0.8 T) induced by an external electromagnet (G MH X 025, Magnet-Schultz GmbH). 12 electromagnets were arranged like a 12 well plate (one per well) and anchored on an aluminum plate (18 cm x 18cm x 0.8 cm) with an incorporated cooling circuit. Solenoids were connected with a switching power supply (PSP 1803, Voltcraft) and controlled by a computer. Cyclic deformation was obtained using LabView (Version 8.2) for regulating power on/off and interval time, respectively. Constant temperature (37°C) of the actuating device was attained using a bath and circulation thermostat (polystat cc3, Huber Kältemaschinenbau GmbH) connected to water cooling system. Equilibrated temperature of the electromagnets was obtained with the aid of a programmed ramp function of the bath and circulation thermostat. When electromagnets were activated the cooling medium was tempered to 32°C (experimentally determined) to compensate the generated heat from the electromagnets. That way, the cell culture medium was always kept at a constant temperature of 37°C within a humidified incubator (data not shown, controlled with an infrared thermometer (Scantemp pro 440)). The specific time interval (2 seconds on, 25 seconds off) was adjusted to stimulate the seeded hMSC on hydrogel scaffolds for 2 x 30 min within 3 h per day (5 weeks, total cycles: 4666) when the influence of scaffold type or chondrogenic medium composition was investigated. 

For the impact of mechanical stimulation at different intensities regarding to differentiation, the stimulus was performed in 30 min cycles every 1.5 h for 8 h per day during 3 weeks (daytime activity, total number of cycles = 12600, 2016 and 672 respectively) for each group (n = 3). Cell culture samples were taken after 1, 2 and 3 weeks, respectively.

### Statistical analysis

All quantitative data are presented as average ± standard deviation. Medium composition, biochemical and mechanical stimulation parameters of chondrogenic differentiation after 5 weeks of culture compared to the control were evaluated by One-way ANOVA (SPSS, 19.0.0). Bonferroni corrections were used to account for multiple comparisons. Differences between groups of p < 0.05 were considered as statistically significant. 

## Results

### Magnetic hydrogels of controlled flexibility

The surface of biochemically inert carbon-coated metal nanomagnets (C/Co, Figure 2a) was chemically functionalized with covalently bound vinyl groups (Supporting Information, Figure S1 in File S1) and crosslinked into a hydrogel polymer backbone[32]. (See Supporting Information, Table S1 and Figure S2 in File S1). The produced hydrogels show a water content close to the one of most mammalian tissues[32]. The here used metal nanomagnets with high saturation magnetization (162 emu g-1)[33] allows rapid and controlled deformation. Analysis (SEM, Figure 2b) of hydrogel cryosections confirmed a homogeneous distribution and porous structure (~ 10 µm), essential for good cell adherence[26]. Elasticity measurements resulted in 21 ± 6 kPa (Supporting Information, Figure S3 in File S1) which is favorable for chondrogenesis[25].

### Magnetic-force controlled stimulation and chondrogenesis

**Figure 3:**
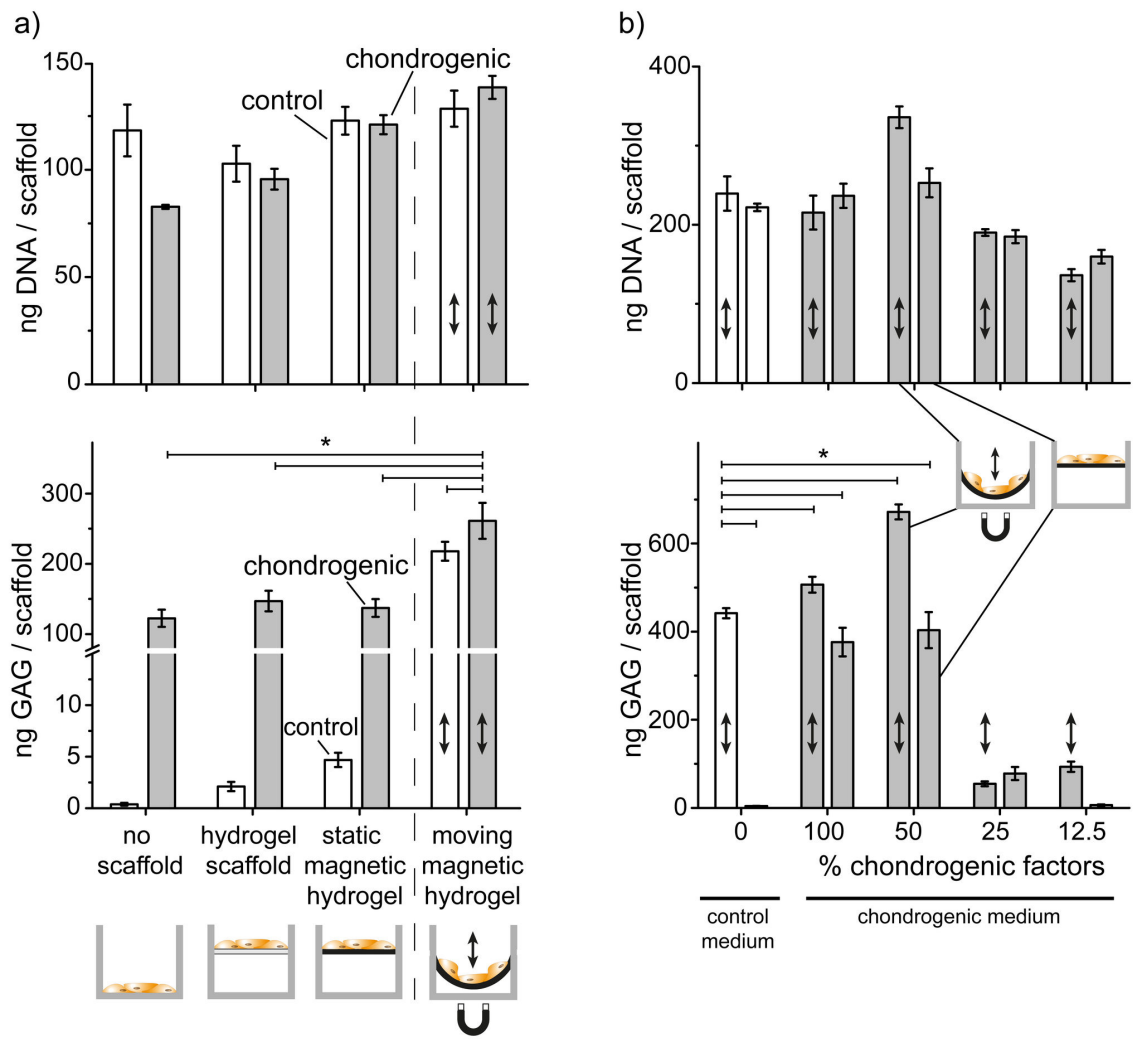
Mechanical stimulation induced chondrogenesis. a) Cell numbers (DNA amount per scaffold) confirmed good cell expansion and growth. Below is the glycosaminoglycan (GAG) deposition per scaffold over a period of 5 weeks. Control medium (white bars) and chondrogenic medium (grey bars) were applied on cells seeded into either tissue culture plate (no scaffold), hydrogel scaffold (no nanomagnets, i.e. no movement is possible) or magnetic hydrogel. Mechanical stimulation (arrow) triggered higher GAG deposition. b) Comparable DNA amount indicated good cell growth for cells seeded into magnetic hydrogels with both medium types and no negative effects from mechanical stimulation. GAG deposition using diluted chondrogenic (grey) versus control medium (white bars). Mechanically stimulated hMSC in control medium showed comparable GAG deposition as in standard chondrogenic medium under magnetic actuation (indicated by ↕). * p < 0.01 cells cultured with control medium under mechanical stimulation versus non stimulated and mechanically stimulated hydrogel using both cell culture media.

Movement in cell cultures has a long tradition based on mechanically connected systems to distort a specific culture surface[12,34]. For very porous, soft and elastic materials, however, such design is unfavorable[25] and leads to scaffold distortions. Binding nano-sized metal magnets to the polymer strands of soft and flexible hydrogels, however, allows in principle to apply a force at each polymer strand, since an external magnetic field will pull each metal nanomagnet, and therefore all polymer strands will move in a similar way. This smooth scaffold deformation is schematically depicted in Figure 3 and Figure S4 in File S1 (Supporting Information). Cells were seeded on magnetic scaffolds (Figure 2d) in control medium or serum-free chondrogenic medium, respectively, and optionally subjected to mechanical stimulation (Supporting Information, Figure S4 in File S1). Cells were cultured for 5 weeks. We further introduced various control experiments: hMSC were cultivated on tissue culture plates (no scaffold), pure hydrogels without nanomagnets and non-stimulated magnetic hydrogels (control experiments to rule out any other influences). Chondrogenesis (i.e. successful differentiation) was quantitatively assessed by measuring sulfated glycosaminoglycan (GAG) deposition, a major component of cartilage extracellular matrix. Importantly, GAG deposition was detected only when cells were cultivated with chondrogenic medium or when cultivated in control medium under mechanical stimulation (Figure 3a). This shows that mechanical stimulation is sufficient to induce and promote chondrogenesis in hMSC. In agreement with the reported literature[35,36], control medium (no movement, no differentiation factors) allows bone marrow derived hMSC to proliferate, but does not induce any differentiation. The absence of GAG formation in control experiments further confirmed that all materials, protocols and treatments used in this work indeed did not significantly influence the hMSC differentiation behavior. The only difference between differentiating and non-differentiating hMSC seeded on the scaffolds used here, was the application of the external magnetic field and the resulting reversible, soft deformation of the scaffold. 

The combination of exogenous chondrogenic factors and mechanical stimulation even amplified the chondrogenesis and resulted in a synergistic effect as evidenced by a significantly higher GAG deposition than non-stimulated scaffolds using chondrogenic medium (p < 0.001; Figure 3a). In contrast, no GAG deposition could be observed for cells cultured in 2D on standard tissue culture plates. Terminal DNA quantification assays confirmed normal hMSC proliferation on tissue culture plates or on control samples in the absence of mechanical stimulation (Figure 3b). A high DNA value obtained for all cell cultivation methods indicated good cell expansion and decreasing LDH activity demonstrated good cytocompatibility (Supporting Information, Figures S5 and S6 in File S1) of the scaffolds and no negative impact by soft, magnetic hydrogel deformation. Nanomagnet-free hydrogels showed comparable cell growth and differentiation behavior if compared to magnetic hydrogels without external magnetic fields (i.e. no mechanical stimulation). This confirms the absence of cytotoxicity, in agreement with earlier studies[37,38].

### Chondrogenesis – The influence of mechanical stimulation combined with different chondrogenic factor concentrations

**Figure 4:**
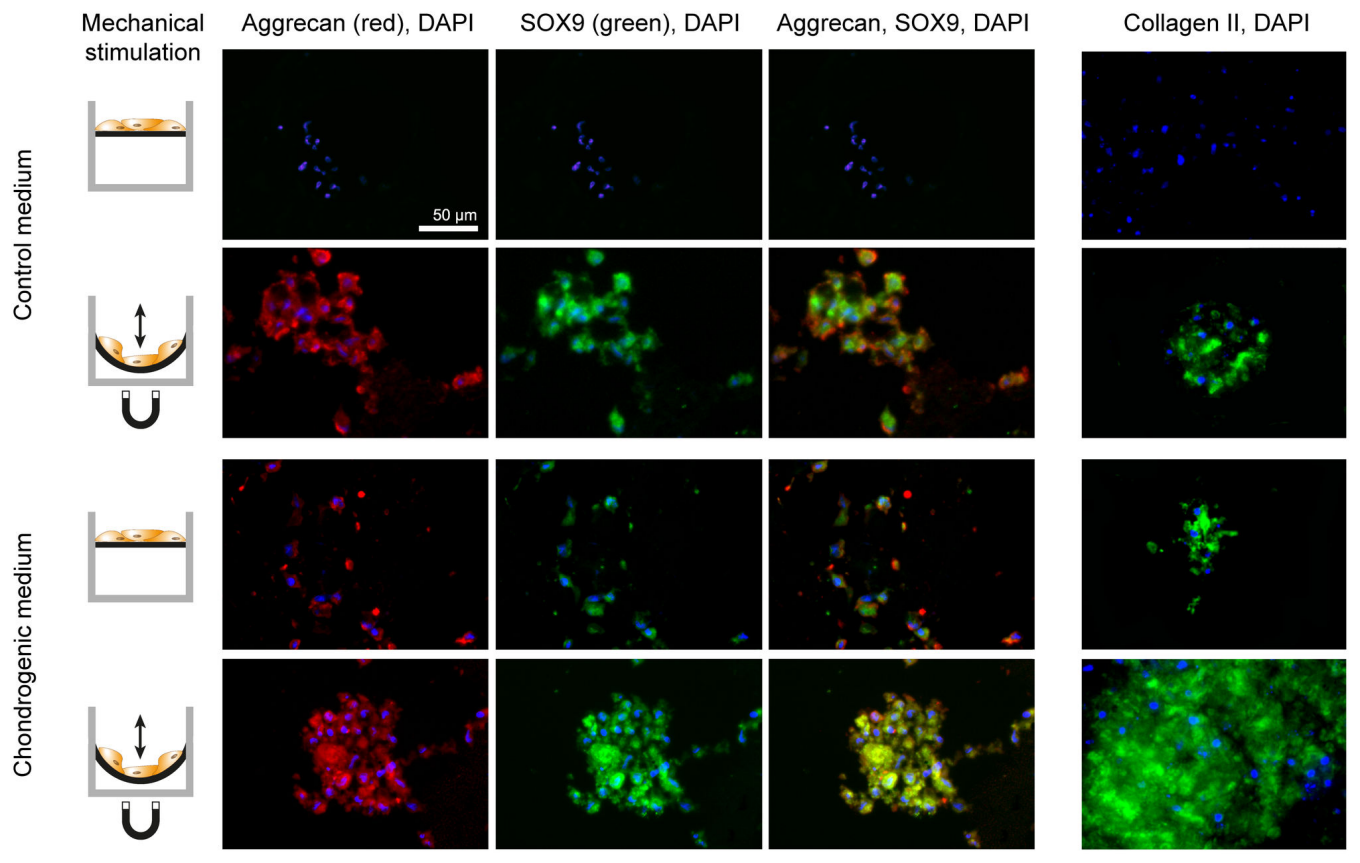
Chondrogenesis on magnetic hydrogels with and without mechanical stimulation. Aggrecan (antibody labeling, red), SOX9 (antibody labeling, green) and Collagen II expression (green) immunohistochemistry of hMSC over a period of 5 weeks. Cell cultures were either not stimulated or underwent repeated mechanical stimulation (left). The role of movement was investigated both in control medium (top rows) and in standard chondrogenic medium (bottom rows). Samples were counterstained with DAPI to make cell nuclei visible. Mechanical stimulation resulted in clear up-regulation of all chondrogenic markers if compared to the non-stimulated control cultures.

Chondrogenesis inducing factors (TGF-β1 and dexamethasone[35,39]) and mechanical stimulation together were investigated at various dilutions and showed a strongly synergistic effect (Figure 3; p < 0.001). The GAG deposition levels indicated a strong influence of mechanical stimulation directing hMSC into chondrogenic lineage even in the absence of any chondrogenesis inducing factors. hMSC cultured at a 50% decreased chondrogenic factors concentration in combination with soft mechanical stimulation displayed a significantly higher degree of chondrogenic differentiation (p < 0.001, Figure 3) compared to standard chondrogenic differentiation medium (classical, non-stimulated cell culture conditions). In addition, chondrogenesis was quantitatively characterized beyond GAG deposition by immunostaining of the cartilage specific proteoglycan core protein aggrecan[40], collagen type II and transcription factor SOX9[41] (Figure 4) providing an independent confirmation of our results. The effects of exposure to both low concentrations of differentiation factor and mechanical stimulation (Figure 3) are complex and require more detailed studies.

### Chondrogenesis – The impact of stimulation frequencies

**Figure 5:**
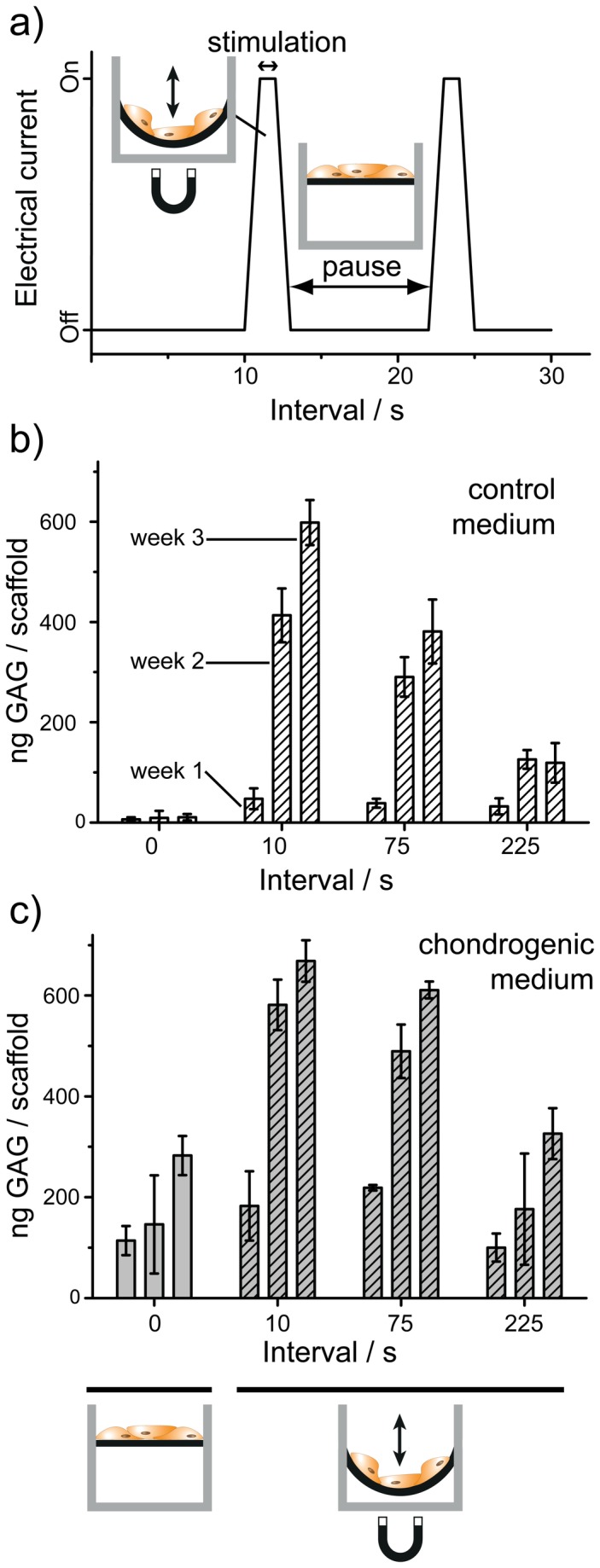
Frequency dependent hMSC differentiation. Mechanical stimulation frequency influences the differentiation and formation of tissue-typical extracellular matrix (amount of GAG formed) in both control and chondrogenic medium. Cells were pressure-free stretched on soft scaffolds for 2 seconds (stimulation period). Non-moved scaffolds (left) served as additional controls. The amount of GAG deposition indicated differentiation on all mechanical stimulated scaffolds particularly at high frequency. This behavior shows that mechanical soft movement follows a dose-effect type response similar to a classical response of specific cells to a given biochemical factor.

The impact of different mechanical stimulation intensities were investigated with cells cultured in medium with reduced chondrogenic factors (50%) and with control medium. The external magnetic field pulse was kept constant in all groups for 2 seconds. The break intervals (electromagnets = off) were set to 10, 75 and 225 seconds (Figure 5). Non-stimulated magnetic hydrogel scaffolds (no movements) served as control. After only 3 weeks of cultivation and stimulation, the amount of GAG deposition had reached at least the same level as described above with less actuation and longer cultivation time (5 weeks). hMSC cultivated with control medium and a break interval of 10 seconds showed almost the same amount of GAG deposition compared to cells cultured in chondrogenic medium (Figure 5). Mechanical stimulation increased the chondrogenic lineage considerably. Fluorescence microscopy analysis again confirmed the biochemical assay results and large, correctly differentiated cell assemblies were observed for cultures in control medium stimulated with 10 seconds break intervals (Supporting Information, Figure S7 in File S1). DNA assays (Supporting Information, Figure S8 in File S1) revealed no negative effect on proliferation when the stimulation frequency was increased and the obtained results were again in line with the controls and previous studies.

## Discussion

Mesenchymal stem cells in healthy and diseased cartilage retain the potential to regenerate tissue[40,42]. While these multi-potent cells have been investigated in tissue engineering of cartilage or bone[4,43-45], our results suggest the possibility of purely mechanical, and therefore local stem cell differentiation. At present, most hMSC studies make use of commercially available or self-designed chondrogenic differentiation mediums with TGF-β, BMP and dexamethasone as key components to chemically induce hMSC differentiation into cartilage associated cells. This obviously creates homogeneous differentiation conditions all over a cell/tissue culture and all structuring must happen through introduction of pre-shaped scaffolds. 

Stem cell differentiation is a combination of intrinsic genetic programs, static factors and microenvironment[46]. Stem cells then dynamically respond and actively modify the properties of their environment by synthesizing or degrading the extracellular matrix, secreting cytokines, and communicating with other cells and matrix by molecular and physical signals[47,48]. All these interactions, however, are based on diffusive processes, or existing organization within a tissue segment and well explains how complex cell assemblies remain stable. This current understanding, however, is unable to explain how large area tissue regeneration is influenced through movement. Our study demonstrates that soft movement in the correctly flexible environment indeed strongly directs hMSC differentiation. Our result is further in line with fundamental clinical observations such as the fact that purely physical stimulation is highly efficient in the treatment of arthritis[49], or, more recently, that mechanical loading alone, without specific growth factors, induces cell alignment and the accumulation of ligament-specific cells in tendons[24]. 

The possibility to use pressure-free movement in the present study has allowed mimicking early embryonic conditions where the development of musculoskeletal apparatus is linked to healthy embryonic movement and where corresponding stem cells differentiate into numerous lineages and highly organized tissues[41].

Our finding that mechanical stimulation alone, without any chondrogenic differentiation factors, can induce chondrogenesis and cartilage-type tissue formation confirms the initial hypothesis that tissue hierarchy cannot be explained on the basis of diffusive processes and polarity alone. Movement induced differentiation, in this regard, is a form of function-driven tissue specialization, where the regenerating tissues execute a function and thereby locally define specific cell’s biological needs and specialization. Mechanical signals have long been shown to trigger cell-surface stretch receptors and adhesion sites, resulting in activation of genes responsible for the synthesis and secretion of extracellular cartilage components[50]. This is in line with the dose-response type correlation between the mechanical stimulation and GAG expression. An alternative explanation based on movement induced fluid flow and increased mass transfer (nutrients or oxygen) as key reasons for improved chondrogenic differentiation can be rejected based on the here used small tissue size (single layer of cells; low metabolic activity) with respect to the available surface for gas and nutrient exchange, and the use of a highly porous substrate. Interestingly, our study is related to the observation that blood flow (also a dynamic component) during embryogenesis has recently been identified as a conserved regulator for hematopoietic stem cell (HSC) formation[51]. 

Another potential discussion point, namely the use of fetal bovine serum (FBS) in standard cell culture medium, can be answered based on the observation that all non-moved control experiments also contained the same amount and batch of bovine serum. FBS was indeed suggested to have an influence on hMSC differentiation[52] but this was not observed during this study. The effect of low frequency electromagnetic fields was suggested to have a positive effect on the chondrogenic differentiation[53]. The controls used here, i.e. hydrogels without nanomagnets in the presence of the same electromagnetic fields did not show any differentiation. This again proves that actual soft movement is indeed the critical stimulus to differentiation even in the absence of any chemical signals or factors.

For all parts of the study, normal proliferation was observed and the absence of cytotoxitiy confirmed excellent cytocompatibility of the used chemically stable and reliably bound nanomagnets. Immunohistochemistry underlined the biochemical assay’s results. Moreover, cells cultured in standard control medium combined with stimulation showed more of chondrogenic characteristics compared to cells cultivated with chondrogenic medium only. Chondrogenesis was clearly accelerated by mechanical stimulation of hMSC cultured in chondrogenic medium. The here observed effects motivate for further studies: The purely mechanically induced stimulation might be the result of local production of growth and differentiation factors and subsequent self-influencing stimulation. Such mechanism would be in line with many growth factors’ capability to stably bind to the basolateral matrix of the cells. Alternatively, the soft, repetitive mechanical deformation might directly have promoted expression of pro-chondrogenic factors.

The here presented elastic, soft, mechanical stimulation cell culturing device uses the highly magnetic, active part of hydrogel scaffolds to convey force through external application of electromagnetic fields. This simple setup allows the same easy handling (seeding, cultivation, medium exchange) as in standard cell culture plates and opens the way for more in depth studies on the relationship between physical deformation, force and local development of tissue function, particularly for the musculoskeletal apparatus, but also in understanding organ barriers (basal membranes), the role of peristaltic movement in maintaining homeostasis in the intestine, and cardiac regeneration. Simple bioreactors may be constructed for larger, 3D magnetic scaffolds and driven by external magnetic forces to mechanically stimulate functionally correct development of larger tissues. With respect to cartilage regeneration, soft and highly magnetic scaffolds are suggested to combine the role of cell culture supports and actuators in tissue engineering of 3D artificial cartilage to treat osteoarthritis patients with their own bone marrow derived stem cells.

## Supporting Information

File S1 **Additional Materials and Methods, Table S1, Figures S1, S2, S3, S4, S5, S6, S7 and S8.**
(PDF)

## References

[1] Vunjak-Novakovic G, David Scadden T (2011) Biomimetic Platforms for Human Stem. Cell Research - Cell Stem Cell 8: 252-261. doi:10.1016/j.stem.2011.02.014. 21362565 PMC3048803

[2] van Meer G (2005) Cellular lipidomics. EMBO J 24: 3159-3165. doi:10.1038/sj.emboj.7600798. PubMed: 16138081. 16138081 PMC1224688

[3] Gumbiner BM (1996) Cell adhesion: The molecular basis of tissue architecture and morphogenesis. Cell 84: 345-357. doi:10.1016/S0092-8674(00)81279-9. PubMed: 8608588. 8608588

[4] Burdick JA, Vunjak-Novakovic G (2009) Engineered Microenvironments for Controlled Stem. Cell Differentiation - Tissue Eng Part A 15: 205-219. doi:10.1089/ten.tea.2008.0131. 18694293 PMC2716398

[5] Grodzinsky AJ, Levenston ME, Jin M, Frank EH (2000) Cartilage tissue remodeling in response to mechanical forces. Annu Rev Biomed Eng 2: 691-713. doi:10.1146/annurev.bioeng.2.1.691. PubMed: 11701528. 11701528

[6] Kim YJ, Sah RLY, Grodzinsky AJ, Plaas AHK, Sandy JD (1994) Mechanical regulation of cartilage biosynthetic behavior - physical stimuli. Arch Biochem Biophys 311: 1-12. doi:10.1006/abbi.1994.1201. PubMed: 8185305. 8185305

[7] Mauck RL, Soltz MA, Wang CCB, Wong DD, Chao PHG et al. (2000) Functional tissue engineering of articular cartilage through dynamic loading of chondrocyte-seeded agarose gels. J Biomech Eng 122: 252-260. doi:10.1115/1.429656. PubMed: 10923293. 10923293

[8] Nagel-Heyer S, Goepfert C, Feyerabend F, Petersen JP, Adamietz P et al. (2005) Bioreactor cultivation of three-dimensional cartilage-carrier-constructs. Bioprocess Biosyst Eng 27: 273-280. doi:10.1007/s00449-005-0419-z. PubMed: 15928929. 15928929

[9] Li Z, Yao SJ, Alini M, Stoddart MJ (2010) Chondrogenesis of Human Bone Marrow Mesenchymal Stem Cells in Fibrin-Polyurethane Composites Is Modulated by Frequency and Amplitude of Dynamic Compression and Shear. Stress - Tissue Eng Part A 16: 575-584. doi:10.1089/ten.tea.2009.0262. 19737049

[10] Rogers BA, Murphy CL, Cannon SR, Briggs TWR (2006) Topographical variation in glycosaminoglycan content in human articular cartilage. J Bone Joint Surg Br 88B: 1670-1674. PubMed: 17159186. 10.1302/0301-620X.88B12.18132 17159186

[11] Masuda T, Takahashi I, Anada T, Arai F, Fukuda T et al. (2008) Development of a cell culture system loading cyclic mechanical strain to chondrogenic cells. J Biotechnol 133: 231-238. doi:10.1016/j.jbiotec.2007.08.007. PubMed: 17904677. 17904677

[12] Grossi A, Yadav K, Lawson MA (2007) Mechanical stimulation increases proliferation, differentiation and protein expression in culture: Stimulation effects are substrate dependent. J Biomech 40: 3354-3362. doi:10.1016/j.jbiomech.2007.05.007. PubMed: 17582421. 17582421

[13] Finger AR, Sargent CY, Dulaney KO, Bernacki SH, Loboa EG (2007) Differential effects on messenger ribonucleic acid expression by bone marrow-derived human mesenchymal stem cells seeded in agarose constructs due to ramped and steady applications of cyclic hydrostatic pressure. Tissue Eng 13: 1151-1158. doi:10.1089/ten.2006.0290. PubMed: 17518710. 17518710

[14] Hoffman BD, Massiera G, Van Citters KM, Crocker JC (2006) The consensus mechanics of cultured mammalian cells. Proc Natl Acad Sci U S A 103: 10259-10264. doi:10.1073/pnas.0510348103. PubMed: 16793927. 16793927 PMC1502445

[15] Langelaan MLP, Boonen KJM, Rosaria-Chak KY, van der Schaft DWJ, Post MJ et al. (2011) Advanced maturation by electrical stimulation: Differences in response between C2C12 and primary muscle progenitor cells. J Tissue Eng Regen Med 5: 529-539. doi:10.1002/term.345. PubMed: 21695794. 21695794

[16] Lee KBL, Hui JHP, Song IC, Ardany L, Lee EH (2007) Injectable mesenchymal stem cell therapy for large cartilage defects - A porcine model. Stem Cells 25: 2964-2971. doi:10.1634/stemcells.2006-0311. PubMed: 17656639. 17656639

[17] Park H, Temenoff JS, Tabata Y, Caplan AI, Mikos AG (2007) Injectable biodegradable hydrogel composites for rabbit marrow mesenchymal stem cell and growth factor delivery for cartilage tissue engineering. Biomaterials 28: 3217-3227. doi:10.1016/j.biomaterials.2007.03.030. PubMed: 17445882. 17445882 PMC2964378

[18] Potier E, Noailly J, Ito K (2010) Directing bone marrow-derived stromal cell function with mechanics. J Biomech 43: 807-817. doi:10.1016/j.jbiomech.2009.11.019. PubMed: 19962149. 19962149

[19] Robling AG, Hinant FM, Burr DB, Turner CH (2002) Improved bone structure and strength after long-term mechanical loading is greatest if loading is separated into short bouts. J Bone Miner Res 17: 1545-1554. doi:10.1359/jbmr.2002.17.8.1545. PubMed: 12162508. 12162508

[20] Hunziker EB, Kapfinger E, Geiss J (2007) The structural architecture of adult mammalian articular cartilage evolves by a synchronized process of tissue resorption and neoformation during postnatal development. Osteoarthritis Cartilage 15: 403-413. doi:10.1016/j.joca.2006.09.010. PubMed: 17098451. 17098451

[21] Roodenburg PJ, Wladimiroff JW, van Es A, Prechtl HFR (1991) Classification and quantitative aspects of fetal movements during the 2nd-half of normal-pregnancy. Early Hum Dev 25: 19-35. doi:10.1016/0378-3782(91)90203-F. PubMed: 2055173. 2055173

[22] Schilling F (1973) Reflex dystrophy and dystrophic pseudoarthritis of lower limbs. Z Rheumaforsch 32: 375-384. PubMed: 4129809. 4129809

[23] Ohta A, Yamaguchi M, Kaneoka H, Nagayoshi T, Hiida M (1987) Adult stills disease - Review of 228 cases from the literature. J Rheumatol 14: 1139-1146. PubMed: 3325642. 3325642

[24] Altman GH, Horan RL, Martin I, Farhadi J, Stark PRH et al. (2001) Cell differentiation by mechanical stress. FASEB J 15: 270-272. doi:10.1096/fj.00-0170hyp. PubMed: 11149915. 11772952

[25] Engler AJ, Sen S, Sweeney HL, Discher DE (2006) Matrix elasticity directs stem cell lineage specification. Cell 126: 677-689. doi:10.1016/j.cell.2006.06.044. PubMed: 16923388. 16923388

[26] Trappmann B, Gautrot JE, Connelly JT, Strange DGT, Li Y et al. (2012) Extracellular-matrix tethering regulates stem-cell fate. Nat Mater 11: 642–649. doi:10.1038/nmat3339. PubMed: 22635042. 22635042

[27] Caplan AI (1991) Mesenchymal stem-cells. J Orthop Res 9: 641-650. doi:10.1002/jor.1100090504. PubMed: 1870029. 1870029

[28] Pittenger MF, Mackay AM, Beck SC, Jaiswal RK, Douglas R et al. (1999) Multilineage potential of adult human mesenchymal stem cells. Science 284: 143-147. doi:10.1126/science.284.5411.143. PubMed: 10102814. 10102814

[29] Prockop DJ (1997) Marrow stromal cells as steam cells for nonhematopoietic tissues. Science 276: 71-74. doi:10.1126/science.276.5309.71. PubMed: 9082988. 9082988

[30] Puetzer JL, Petitte JN, Loboa EG (2010) Comparative Review of Growth Factors for Induction of Three-Dimensional In Vitro Chondrogenesis in Human Mesenchymal Stem Cells Isolated from Bone Marrow and Adipose Tissue. Tissue Eng Part B Rev 16: 435-444. doi:10.1089/ten.tec.2009.0247. PubMed: 20196646. 20196646

[31] Clevers H (2006) Wnt/beta-catenin signaling in development and disease. Cell 127: 469-480. doi:10.1016/j.cell.2006.10.018. PubMed: 17081971. 17081971

[32] Fuhrer R, Athanassiou EK, Luechinger NA, Stark WJ (2009) Crosslinking Metal Nanoparticles into the Polymer Backbone of Hydrogels Enables Preparation of Soft, Magnetic Field-Driven Actuators with Muscle-Like Flexibility. Small 5: 383-388. doi:10.1002/smll.200801091. PubMed: 19180549. 19180549

[33] Grass RN, Stark WJ (2006) Gas phase synthesis of fcc-cobalt nanoparticles. J Mater Chem 16: 1825-1830. doi:10.1039/b601013j.

[34] Michalopoulos E, Knight RL, Korossis S, Kearney JN, Fisher J et al. (2012) Development of Methods for Studying the Differentiation of Human Mesenchymal Stem Cells Under Cyclic Compressive Strain. Tissue Eng Part C Methods 18: 252-262. doi:10.1089/ten.tea.2011.0142. PubMed: 22047076. 22047076 PMC3311877

[35] Reger RL, Tucker AH, Wolfe MR (2008) Differentiation and characterization of human MSCs. In: Prockop DJ Phinney DG Bunnell BA Methods in Molecular Biology. Humana Press Inc, Totowa, USA pp. 93-107. 10.1007/978-1-60327-169-1_7 18370086

[36] Meinel L, Karageorgiou V, Fajardo R, Snyder B, Shinde-Patil V et al. (2004) Bone tissue engineering using human mesenchymal stem cells: Effects of scaffold material and medium flow. Ann Biomed Eng 32: 112-122. doi:10.1023/B:ABME.0000007796.48329.b4. PubMed: 14964727. 14964727

[37] Herrmann IK, Urner M, Koehler FM, Hasler M, Roth-Z'Graggen B et al. (2010) Blood Purification Using Functionalized Core/Shell Nanomagnets. Small 6: 1388-1392. doi:10.1002/smll.201000438. PubMed: 20564487. 20564487

[38] Weber W, Lienhart C, Baba MDE, Grass RN, Kohler T et al. (2009) Magnet-guided transduction of mammalian cells and mice using engineered magnetic lentiviral particles. J Biotechnol 141: 118-122. doi:10.1016/j.jbiotec.2009.02.023. PubMed: 19433214. 19433214

[39] Shintani N, Hunziker EB (2011) Differential effects of dexamethasone on the chondrogenesis of mesenchymal stromal cells: influence of microenvironment, tissue origin and growth factor. Eur Cell Mater 22: 302-320. PubMed: 22116649. 22116649 10.22203/ecm.v022a23

[40] Johnson K, Zhu S, Tremblay MS, Payette JN, Wang J et al. (2012) A Stem Cell-Based Approach to Cartilage Repair. Science 336: 717-721. doi:10.1126/science.1215157. PubMed: 22491093. 22491093

[41] Bi WM, Deng JM, Zhang ZP, Behringer RR, de Crombrugghe B (1999) Sox9 is required for cartilage formation. Nat Genet 22: 85-89. doi:10.1038/8792. PubMed: 10319868. 10319868

[42] Magne D, Vinatier C, Julien M, Weiss P, Guicheux J (2005) Mesenchymal stem cell therapy to rebuild cartilage. Trends Mol Med 11: 519-526. doi:10.1016/j.molmed.2005.09.002. PubMed: 16213191. 16213191

[43] Lutolf MP, Gilbert PM, Blau HM (2009) Designing materials to direct stem-cell fate. Nature 462: 433-441. doi:10.1038/nature08602. PubMed: 19940913. 19940913 PMC2908011

[44] Mitragotri S, Lahann J (2009) Physical approaches to biomaterial design. Nat Mater 8: 15-23. doi:10.1038/nmat2344. PubMed: 19096389. 19096389 PMC2793340

[45] Coburn JM, Gibson M, Monagle S, Patterson Z, Elisseeff JH (2012) Bioinspired nanofibers support chondrogenesis for articular cartilage repair. Proc Natl Acad Sci U S A 109: 10012-10017. doi:10.1073/pnas.1121605109. PubMed: 22665791. 22665791 PMC3382486

[46] Galle J, Bader A, Hepp P, Grill W, Fuchs B et al. (2010) Mesenchymal Stem Cells in Cartilage Repair: State of the Art and Methods to monitor Cell Growth, Differentiation and Cartilage Regeneration. Curr Med Chem 17: 2274-2291. doi:10.2174/092986710791331095. PubMed: 20459378. 20459378

[47] Place ES, Evans ND, Stevens MM (2009) Complexity in biomaterials for tissue engineering. Nat Mater 8: 457-470. doi:10.1038/nmat2441. PubMed: 19458646. 19458646

[48] Even-Ram S, Artym V, Yamada KM (2006) Matrix control of stem cell fate. Cell 126: 645-647. doi:10.1016/j.cell.2006.08.008. PubMed: 16923382. 16923382

[49] Snow WB (1943) The relation of physical therapy to arthritis. N Engl J Med 229: 959-965. doi:10.1056/NEJM194312232292601.

[50] Toyoda T, Matsumoto H, Fujikawa K, Saito S, Inoue K (1998) Tensile load and the metabolism of anterior cruciate ligament cells. Clin Orthop Relat Res: 247-255. PubMed: 9728181. 10.1097/00003086-199808000-00029 9728181

[51] North TE, Goessling W, Peeters M, Li PL, Ceol C et al. (2009) Hematopoietic Stem Cell Development Is Dependent on Blood Flow. Cell 137: 736-748. doi:10.1016/j.cell.2009.04.023. PubMed: 19450519. 19450519 PMC2722870

[52] Gong Z, Calkins G, Cheng EC, Krause D, Niklason LE (2009) Influence of culture medium on smooth muscle cell differentiation from human bone marrow-derived mesenchymal stem cells. Tissue Eng Part A 15: 319-330. doi:10.1089/ten.tea.2008.0161. PubMed: 19115826. 19115826 PMC2716410

[53] Mayer-Wagner S, Passberger A, Sievers B, Aigner J, Summer B et al. (2011) Effects of Low Frequency Electromagnetic Fields on the Chondrogenic Differentiation of Human Mesenchymal Stem Cells. Bioelectromagnetics 32: 283-290. doi:10.1002/bem.20633. PubMed: 21452358. 21452358

