# Supplementary material for: Pressureless Mechanical Induction of Stem Cell Differentiation Is Dose and Frequency Dependent
Source: PLoS One. 2013 Nov 21;8(11):e81362. doi: 10.1371/journal.pone.0081362 (PMC3836961; doi:10.1371/journal.pone.0081362)
Supplement: File S1 — Additional Materials and Methods, Table S1, Figures S1, S2, S3, S4, S5, S6, S7 and S8. (PDF) [file pone.0081362.s001.pdf]

Supporting Information for

Pressureless mechanical induction of stem cell differentiation is dose and frequency dependent

Roland Fuhrer[1], Sandra Hofmann[2], Nora Hild[1], Jolanda R. Vetsch[2], Inge K. Herrmann[3], Robert N. Grass[1], Wendelin J. Stark[1]*

[1] Institute for Chemical and Bioengineering, ETH Zurich, Zurich, Switzerland

[2] Institute for Biomechanics, ETH Zurich, Zurich, Switzerland

[3] Institute of Anaesthesiology, University Hospital Zurich, Zurich, Switzerland

* E-mail: wendelin.stark@chem.ethz.ch

Materials and Methods

The production of the here used chemically functionalized nanomagnets is already described elsewhere [1].

Pretreatment of nanomagnets

Carbon-coated metal nanomagnets (C/Co, Turbobeads LLC, Switzerland) were treated in aqueous 1 wt% HCl (Fluka, puriss) using deionized water (Millipore) until the pH remained stable, and washed three times with water (Millipore) and acetone (puriss, Fluka), respectively. In between nanomagnets were suspended by sonication (Sonorex RK 106, Bandelin) for 5 min and recovered with the aid a commercial neodymium based magnet (N42, Q-40-40-20-N, Webcraft GmbH) at the glass wall. At the end, the nanomagnets were dried *in vacuo* at 50°C.

Nanomagnets 4-Iodo-phenyl functionalization

C/Co (10 g) were suspended in 60 mL of a precooled solution of sodium nitrite (11.5 mmol, puriss p.a. ≥99%, Fluka) using an ultrasonic bath. The 4-iodobenzene diazonium ion was prepared *in-situ* by adding 100 mL of a 0°C cooled solution of 4-iodoaniline (7.5 mmol, 98%, Aldrich) and hydrochloric acid (HCl conc., 3 mL, puriss p.a. ≥36.5%, Fluka). The reaction mixture was alternately left for 5 min in ultrasonic or ice bath to react for total 30 min. The nanomagnets were collected, washed three times each with water, hexane, ethyl-acetate and acetone (all solvents: puriss, Fluka), and resuspended at each step, as described above, then dried *in vacuo* at 50°C.

Nanomagnets 4-vinyl-biphenyl functionalization

Using a Suzuki coupling reaction [2], 4-iodo-phenyl functionalized nanomagnets (5 g), 4-vinylbenzene boronic acid (8.13 mmol, 98%, ABCR GmbH), palladium(II) acetate (0.38 mmol, 98%, Aldrich), triphenylphosphine (1.13 mmol, puriss ≥98%, Fluka) and sodium carbonate (8.96 mmol, pro analysi,

Merck) were added under nitrogen flow to degassed 1-propanol (32 mL, 99.9%, Alfa Aesar)/deionized water (32 mL, Millipore). After complete dissolution, the reaction mixture was stirred for 16 h at 95°C under continuous purging with nitrogen. After cooling, functionalized nanomagnets were washed and dried as described above.

Chemical characterization of functionalized nanomagnets

Fourier transform infrared spectroscopy (FTIR) measurements of nanomagnets were done in potassium bromide (5 wt% particles, KBr, ≥99% trace metals basis, Sigma-Aldrich) using a Tensor 27 Spectrometer (Bruker Optics, equipped with a diffuse reflectance accessory, DiffusIR, Pike technologies). Bare metal nanomagnets were taken as a background to improve the spectrum quality. Element microanalysis (LECO CHN-900) was used to acquire quantitative confirmation of completion of the reaction and chemical functionalization yield. Transmission electron microscopy (TEM) images were recorded on a CM 12 (Philips, Tungsten filament, operated at 100 kV, point resolution 10 Å). Nanomagnets were deposited onto a carbon foil supported by a copper grid.

Magnetic hydrogel synthesis

Appropriate amounts of 2-hydroxy-ethyl-methacrylate (HEMA, 5.1 mL, puriss. ≥99%, Fluka), ethylene glycol dimethacrylate (EGDMA, 4.9 µl, purum ≥97%, Fluka) and styrene maleic anhydride copolymer (SMA® 1000H, 0.75 g, 36%, Sartomer) solution were mixed in water (25 mL, Millipore). Subsequently, 4-vinylbiphenyl functionalized nanomagnets (5 g) were suspended in the mixture using an ultrasonic probe (UP400S, 24 kHz, Hielscher GmbH) during 5 min. After addition of a rheology additive (BYK® 420, 0.782 g, Brenntag Schweizerhall AG), tetramethylethylenediamine (TMEDA, 31.9 µl, 99%, Sigma Aldrich) and ammonium persulfate (APS, 205 mg, 99+%, DNAse, RNAse and protease free, for molecular biology, Acros Organics) the mixture was processed again with the ultrasonic probe for 5 min. The reaction mixture was then poured into an electrophoresis casting mold

(gel casting mold, kuroGEL Midi 13) and reacted for 1 h at ambient temperature. The obtained gel was treated with deionized water for 24 h while the water was changed 3 times. The procedure was repeated with phosphate-buffered saline (PBS, pH 7.4, GIBCO) to remove unreacted monomer and nanomagnets and to obtain a stable swelling behavior of the hydrogel. The obtained deep-black magnetic hydrogel (2 mm thickness) was punched out to the desired shape and sterilized in 70% ethanol. Sterile scaffolds were rinsed 3 times with fresh PBS to remove any residual ethanol. A dog-bone like shape of the scaffold was chosen to increase the flexibility and enable a hammock like deformation. This structure allowed for minimization of the required magnetic force.

Characterization of magnetic hydrogel

Magnetic hydrogel discs with 10 mm diameter and 2 mm thickness were investigated regarding their elastic modulus. Incompressibility tests were performed at ambient conditions on an Instron's electromechanical testing system (Instron 5864 equipped with a 10 kN load cell and cross head speed of 2 mm min^{-1}). Hydrogel samples were investigated by scanning electron microscopy on a FEG 1530 Zeiss Gemini after cryosectioning and placing on carbon tape glued to aluminum stubs.

Cell isolation, expansion and characterization:

hMSC isolation from human bone marrow (Lonza Group Ltd) was performed by cell adherence to tissue culture plates as described previously [3,4]. Frozen cell aliquots were resuspended in expansion medium containing Dulbecco's modified eagle medium (DMEM, Invitrogen), 10% fetal bovine serum (FBS, Invitrogen), 1% antibiotic/antimycotic (100x, Invitrogen), 1% non essential amino acids (NEAA, 7.5 mg L^{-1} glycine, 8.9 mg L^{-1} L-alanine, 13.2 mg L^{-1} L-asparagine, 13.3 mg L^{-1} L-aspartic acid, 14.7 mg L^{-1} L-glutamic acid, 11.5 mg L^{-1} L-proline, 10.5 mg L^{-1} L-serine, Invitrogen) and 1 ng L^{-1} basic fibroblast growth factor (bFGF, Invitrogen). The cells were seeded in 175 cm^2 triple flasks at a density of 5 x 10^4 cells cm^{-2}. Adherent cells were allowed to reach approximately 80% confluence and

trypsinized after 6–8 days (trypsin-EDTA, 0.25%, Invitrogen). The third passage (P3) of the cells was used in all conducted studies. For each investigation freshly expanded hMSC were applied. To confirm their mesenchymal character, the cells were characterized at various passages with respect to their proliferative potential, the characteristic expression of surface antigens and the ability to selectively differentiate into chondrogenic or osteogenic lineages in response to environmental stimuli [5].

Scaffold seeding

A homogeneous distribution of seeded cells onto the hydrogel surface was obtained by using small volumes of relatively high concentrated cell suspensions ($2.4 \cdot 10^6$ P3 hMSC mL^{-1}) either in control (DMEM, 10% FBS, 1% antibiotic/antimycotic) or chondrogenic medium (DMEM, 1% antibiotic/antimycotic, 100 nM dexamethasone (water-soluble, cell culture tested, Sigma-Aldrich), 6.25 µg mL^{-1} insulin (bovine pancreas, Sigma-Aldrich), 50 µg mL^{-1} ascorbic acid (≥95%, Sigma-Fine Chemicals), 40 µg mL^{-1} L-proline (≥98.5%, Sigma-Fine Chemicals), 6.25 µg mL^{-1} ITS (BD ITS+ Premix, BD Biosciences) and 10 ng mL^{-1} transforming growth factor beta 1 (rh TGF-β1, R&D Systems, [6]). Precut and sterilized hydrogel scaffolds were fixed with the aid of sterile stainless steel rings in wells of a 12 well plate (non-tissue-culture-treated, Falcon). The lower ring acted as a spacer with 2 mm thickness to allow hydrogel deformation. 250 µL of the respective cell suspension was seeded onto each scaffold and cells were left for 20 min at 5% CO_2 and 37°C to allow adherence on the scaffold surface. An additional 1.75 mL of the respective medium was then gently added to each well. All cell culture experiments were performed in triplicates and the corresponding medium was replaced three times a week.

Cell attachment and viability

Successful cell seeding and adherence was verified by using 4 mM calcein acetoxymethyl ester (calcein AM, Invitrogen) in the corresponding medium for 20 min in a humidified incubator (37°C, 5% CO_2).

Fluorescence images from cells cultivated on scaffolds were immediately obtained using an inverted research microscope equipped with reflected fluorescence system (IX51, Olympus). Cell cytotoxicity was directly measured from the supernatant using a cytotoxicity detection kit measuring the lactate dehydrogenase activity according to the manufacturer instructions (Roche Applied Science).

Cyclical magnetically assisted mechanical stimulation

The vertical motion of the magnetic, soft hydrogel scaffold was controlled by a magnetic field (0.8 T) induced by an external electromagnet (G MH X 025, Magnet-Schultz GmbH). 12 electromagnets were arranged like a 12 well plate (one per well) and anchored on an aluminum plate (18 cm x 18cm x 0.8 cm) with an incorporated cooling circuit. Solenoids were connected with a switching power supply (PSP 1803, Voltcraft) and controlled by a computer. Cyclic deformation was obtained using LabView (Version 8.2) for regulating power on/off and interval time, respectively. Constant temperature (37°C) of the actuating device was attained using a bath and circulation thermostat (polystat cc3, Huber Kältemaschinenbau GmbH) connected to water cooling system. Equilibrated temperature of the electromagnets was obtained with the aid of a programmed ramp function of the bath and circulation thermostat. When electromagnets were activated the cooling medium was tempered to 32°C (experimentally determined) to compensate the generated heat from the electromagnets. That way, the cell culture medium was always kept at a constant temperature of 37°C within a humidified incubator (data not shown, controlled with an infrared thermometer (Scantemp pro 440)). The specific time interval (2 sec on, 25 sec off) was adjusted to stimulate the seeded hMSC on hydrogel scaffolds for 2 x 30 min within 3 h per day (5 weeks, total cycles: 4666) when the influence of scaffold type or chondrogenic medium composition was investigated.

For the impact of mechanical stimulation at different intensities regarding to differentiation, the stimulus was performed in 30 min cycles every 1.5 h for 8 h per day during 3 weeks (daytime activity,

total number of cycles = 12600, 2016 and 672 respectively) for each group (n = 3). Cell culture samples were taken after 1, 2 and 3 weeks, respectively.

Cell expansion

Cell expansion was determined by DNA analysis. At the end of the respective culture period scaffolds were rinsed 3 times with PBS, cut into quarter, weighed and disintegrated using a minibead-beater (Biospec) and two stainless steel balls (6.35 mm, Biospec) in 1 mL 0.2% triton X-100 (BioXtra, Fluka) and 5 mM $MgCl_2$ (BioUltra, for molecular biology, ≥99.0%, Fluka) solution. Quant-iT™ PicoGreen® dsDNA assay (Invitrogen) was used to determine the DNA amount according to the manufacturer's protocol. Samples (n = 3) were measured with a plate reader (Infinite F200, Tecan) in triplicates using black flat-bottom 96 well plates (Nunc) at an excitation / emission wavelength of 480 nm / 530 nm respectively.

Chondrogenic differentiation

At the end of the respective culture period chondrogenic differentiation on the scaffold (cut in quarters) was detected by measuring sulfated glycosaminoglycans (GAG) using 1,9-dimethylenemethylblue [7-9]. Three samples per group were freeze-dried (Alpha 1-2, Martin Christ GmbH) and digested in 1 mL papain solution (500 U mL^{-1}, Worthington) buffered with 14.4 mM L-cystein (BioUltra, ≥99.5% RT, Sigma-Fine Chemicals), 0.1 M disodium hydrogen phosphate (cell culture grade, Sigma-Fine Chemicals) and 0.01 M ethylenediaminetetraacetic acid (EDTA, ≥99%, Sigma Aldrich) for 16 h at 60°C. The amount of GAG was detected in triplicates with a plate reader using transparent 96 well plate (Nunc) at 525 nm with chondroitin sulfate (cell culture tested, Sigma-Fine Chemicals) as standard.

Immunohistochemistry

Scaffold quarters (n = 3) were rinsed with PBS and fixed in 4% neutral buffered formalin (36 h at 4°C), mounted in OCT embedding compound (CellPath) and snap freezed at -196°C. Cryosectioning (Cryo-Star HM 560 MV) was used to cut 6 µm thick scaffold sections which were thaw-mounted onto SuperFrost microscope slides (Microm International AG). For detection of aggrecan an anti-human aggrecan primary antibody (Ig type: goat IgG, R&D Systems) was used and stained with a secondary antibody (Alexa Fluor 488 donkey anti-goat IgG, Invitrogen). Verification of transcription factor SOX9 was provided with NL557 conjugated primary antibody anti-human SOX9 (R&D Systems). After blocking non specific staining sites with buffered bovine serum albumin (30 mg mL^{-1} in PBS, ≥96%, Sigma-Fine Chemicals) primary antibodies were incubated overnight at 4°C and stained with secondary antibody for 30 min at room temperature. Finally, samples were counterstained with dimethyl pimelimidate dihydrochloride (300 nM in PBS, DAPI, Sigma-Fine Chemicals) during 3 min to visualize the cell nuclei. Images were acquired with an Olympus CellR fluorescence microscope and processed (coloring and overlay) with the ImageJ software (1.45S, Wayne Rasband, National Institutes of Health, USA).

Table S1 presents quantitative information about the preparation of vinyl-functionalized carbon coated cobalt nanomagnets.

Table S1. Element analysis for carbon and iodine content.

	C [wt%]	Iodine [wt%]	Loading [mmol g^{-1}]
C/Co[a]	5.6	0.02	-
C/Co-Iodine	8.0	2.7	0.23
C/Co-Vinyl	10.1	0.3	0.24

[a] TurboBeads as prepared nanoparticles.

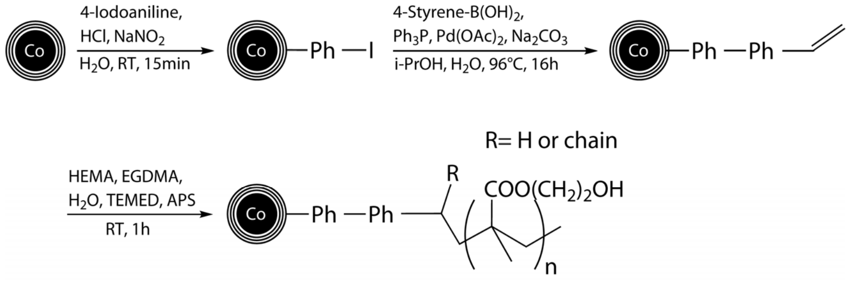

Figure S1. Chemical modification of the nanomagnet surface

Metallic carbon coated cobalt (C/Co) nanomagnets were chemically functionalized using diazonium radical chemistry, Suzuki coupling, and radical polymerization to provide a chemically defined soft scaffold with force anchoring. 2-Hydroxyethyl methacrylate (HEMA) was crosslinked with ethylene glycol dimethacrylate (EGDMA) in water using ammonium persulfate (APS) as an initiator and Tetramethylethylenediamine (TEMED) as catalyst.

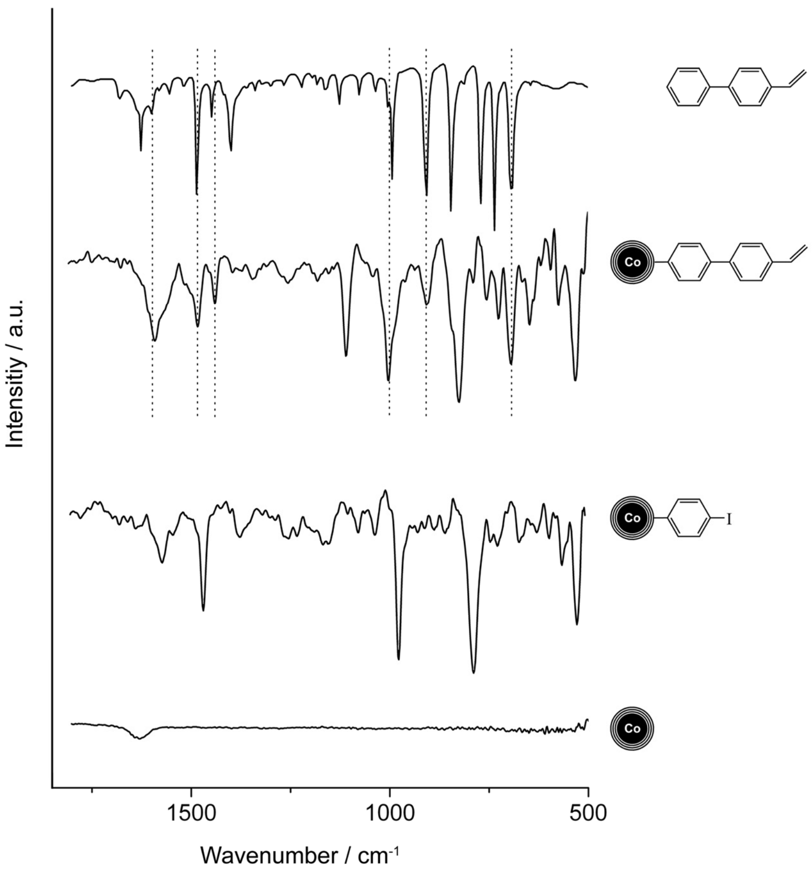

Figure S2. Chemical identity of the carbon shell modifications

Fourier transform infrared spectra of untreated metal nanomagnets C/Co (bottom trace, no peaks) and the subsequent functionalization steps via 4-iodo-phenyl (second trace) and 4-vinylbiphenyl-coated nanomagnets (third trace). A comparison with a related reference material (4-vinyl-biphenyl, forth trace) shows a comparable peak pattern.

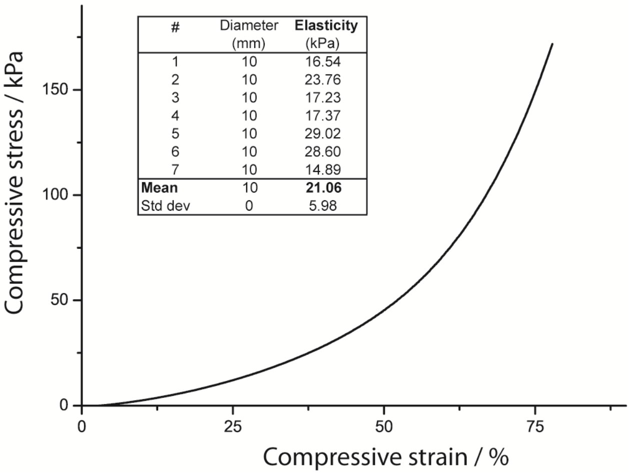

Figure S3. Elasticity measurement of magnetic hydrogel

Using an Instron's electromechanical testing system of the here used magnetic hydrogel showing a stiffness comparable to native human tissue of an anatomically similar origin.

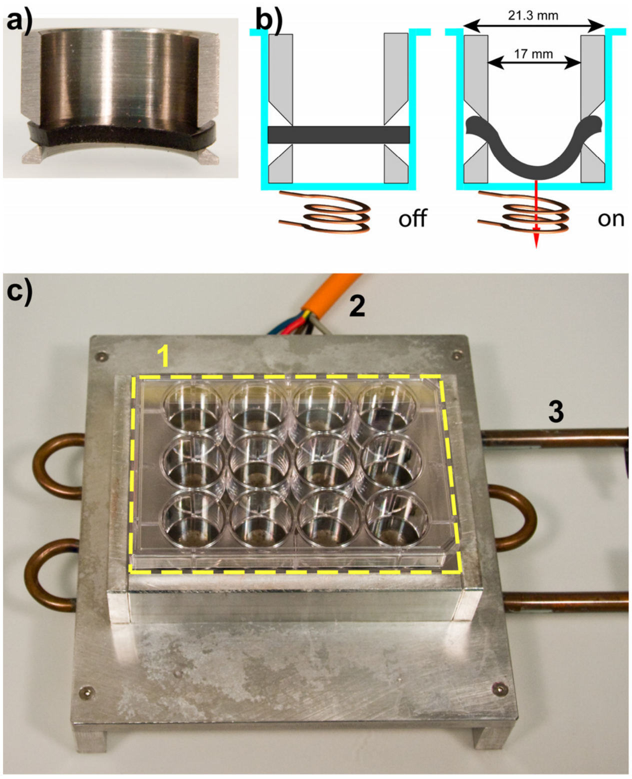

Figure S4. Cell culture on soft and externally magnetic-field driven scaffolds

a) Magnetic hydrogels were fixed between two sterilized stainless steel rings.

b) Copped rings allowed for an easier scaffold deformation when the external magnetic field was turned on.

c) The fixed soft scaffolds were directly placed into wells of a standard 12-well plate (1) which was then positioned onto temperature controlled electromagnets. Solenoids were connected to an external switching power supply (2) and controlled by a computer. Copper tubing (3) and a programmable circulation thermostat maintained constant 37°C in the cell culture medium. The whole setup was then placed in an incubator.

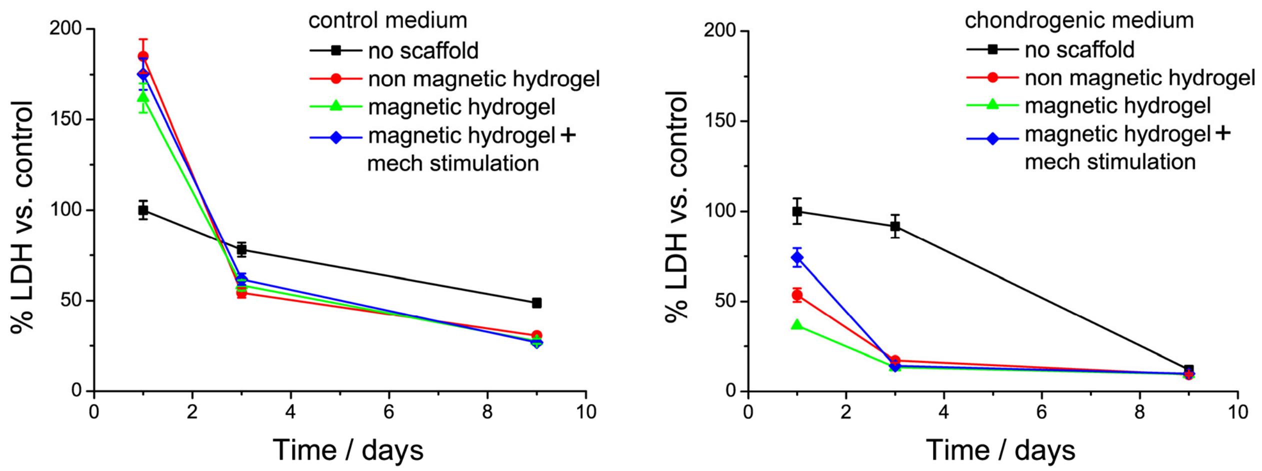

Figure S5. Lactat dehydrogenase (LDH) activity of cultured cells on different scaffold types

Descending LDH release confirmed the absence of cytotoxic effects for all scaffold types. LDH activity, relative to the control (no scaffold), in standard control and chondrogenic medium was investigated with pure hydrogel (no nanomagnets), magnetic hydrogel and mechanically stimulated hydrogel.

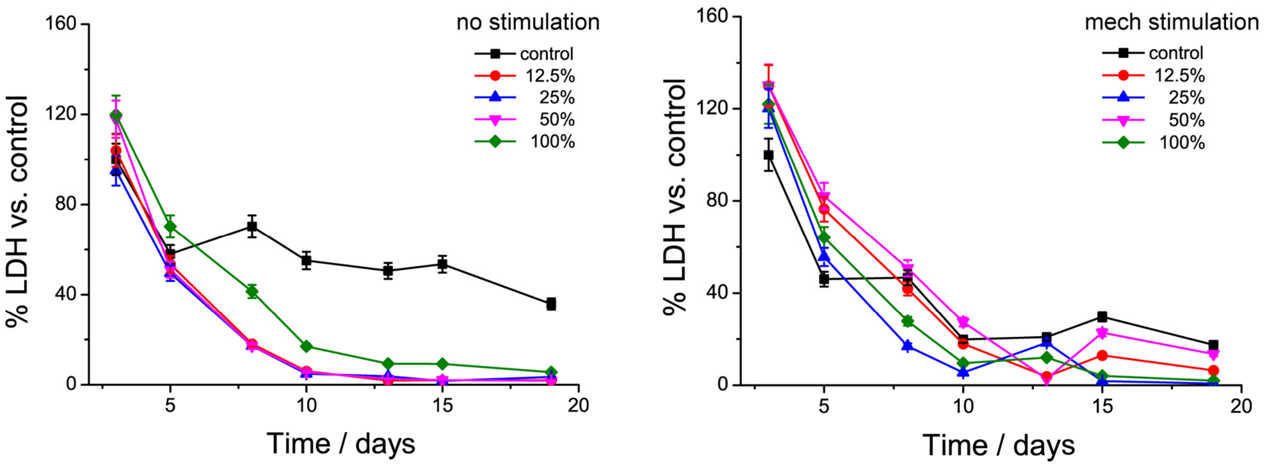

Figure S6. Lactate dehydrogenase (LDH) activity of cells cultured with different medium types

Different medium types i.e. chondrogenic medium, subsequently diluted with serum-free medium to 50%, 25% and 12.5% and standard control medium were used for cell cultivation. The clear decrease of LDH activity over time and coincident high DNA amount (Fig. 3d) underline good cytocompatibility of the magnetic scaffolds even with no mechanical stimulation (left) or magnetic stimulation (right).

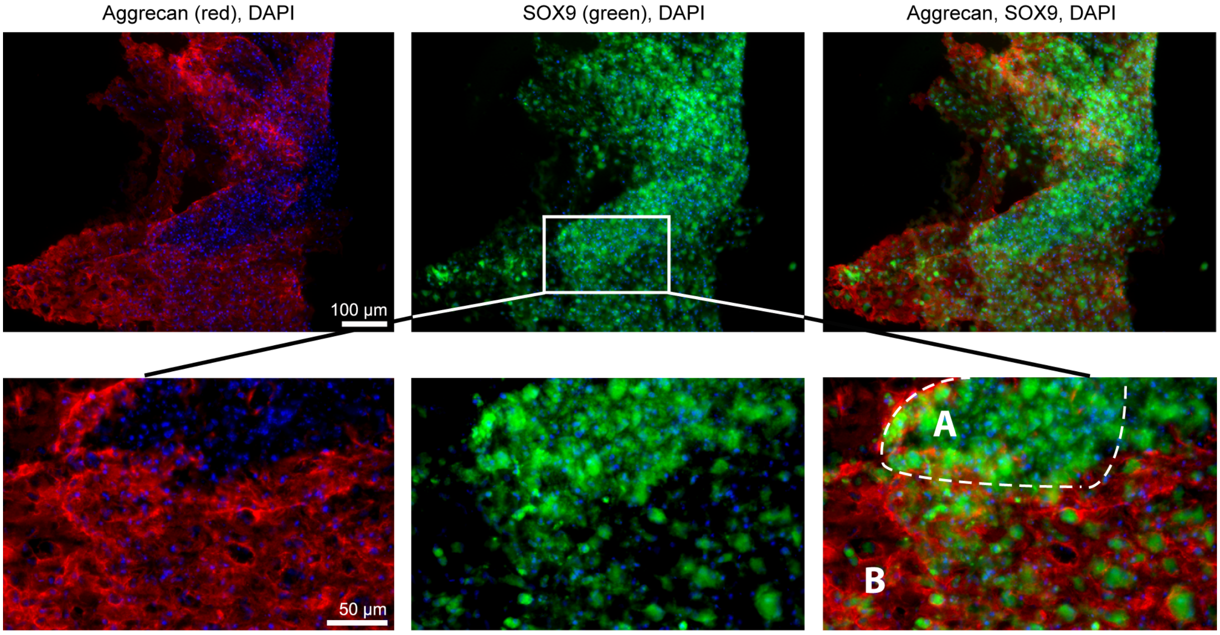

Figure S7. Cartilage specific cell assembly formation

Chondrogenesis through high stimulation frequency in hMSC culture using standard control medium (no differentiation factors added) resulted in sub-millimeter sized cartilage specific cell assemblies over larger areas of the magnetic scaffold. High SOX9 expression (antibody labeling, green) and low aggrecan signaling (antibody labeling, red) indicate ongoing chondrogenic differentiation in region A (right). Differentiation had already taken place in region B indicated through low SOX9 expression and high aggrecan signaling. This micro-tissue level demonstration of purely mechanically induced cartilage formation *in vitro* further confirms the dominant role of movement in stem cell differentiation. All samples were counterstained with DAPI.

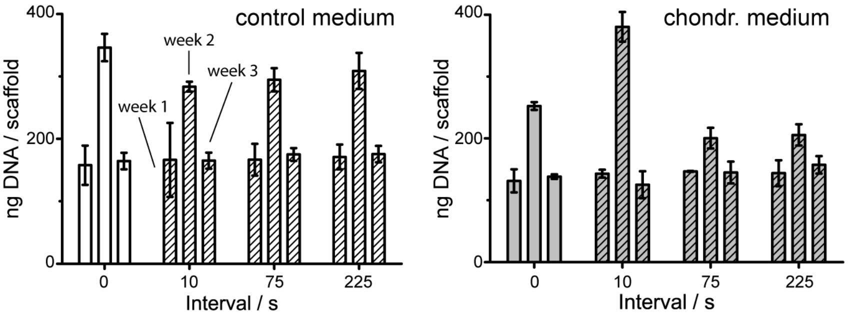

Figure S8. Frequency dependence hMSC differentiation did not affect cell proliferation (DNA content)

Higher frequencies had no negative impact towards cell growth using control (white bars) and chondrogenic medium (grey bars) compared to the control cultures (labeled with 0, control medium, no stimulation). Mechanical stimulation is indicated with patterns.

References

1. Fuhrer R, Athanassiou EK, Luechinger NA, Stark WJ (2009) Crosslinking Metal Nanoparticles into the Polymer Backbone of Hydrogels Enables Preparation of Soft, Magnetic Field-Driven Actuators with Muscle-Like Flexibility. Small 5: 383-388.

2. Tan CG, Grass RN (2008) Suzuki cross-coupling reactions on the surface of carbon-coated cobalt: expanding the applicability of core-shell nano-magnets. Chem Commun: 4297-4299.

3. Meinel AJ, Kubow KE, Klotzsch E, Garcia-Fuentes M, Smith ML, et al. (2009) Optimization strategies for electrospun silk fibroin tissue engineering scaffolds. Biomaterials 30: 3058-3067.

4. Meinel L, Karageorgiou V, Fajardo R, Snyder B, Shinde-Patil V, et al. (2004) Bone tissue engineering using human mesenchymal stem cells: Effects of scaffold material and medium flow. Ann Biomed Eng 32: 112-122.

5. Hofmann S, Hagenmuller H, Koch AM, Muller R, Vunjak-Novakovic G, et al. (2007) Control of in vitro tissue-engineered bone-like structures using human mesenchymal stem cells and porous silk scaffolds. Biomaterials 28: 1152-1162.

6. Puetzer JL, Petitte JN, Loboa EG (2010) Comparative Review of Growth Factors for Induction of Three-Dimensional In Vitro Chondrogenesis in Human Mesenchymal Stem Cells Isolated from Bone Marrow and Adipose Tissue. Tissue Eng Part B Rev 16: 435-444.

7. Farndale RW, Buttle DJ, Barrett AJ (1986) Improved quantitation and discrimination of sulfated glycosaminoglycans by use of dimethylethylene blue. Biochim Biophys Acta 883: 173-177.

8. Hoemann CD (2004) Molecular and Biochemical Assays of Cartilage Components Cartilage and Osteoarthritis. In: Ceuninck F, Sabatini M, Pastoureau P, editors. Methods in Molecular Medicine: Humana Press Inc, Totowa, USA. pp. 127-156.

9. Hofmann S, Knecht S, Langer R, Kaplan DL, Vunjak-Novakovic G, et al. (2006) Cartilage-like tissue engineering using silk scaffolds and mesenchymal stem cells. Tissue Eng 12: 2729-2738.
